# Hyperdiploidy: the longest known, most prevalent, and most enigmatic form of acute lymphoblastic leukemia in children

**DOI:** 10.1038/s41375-022-01720-z

**Published:** 2022-10-20

**Authors:** Oskar A. Haas, Arndt Borkhardt

**Affiliations:** 1 St. Anna Children’s Hospital, Pediatric Clinic, Medical University, Vienna, Austria; 2 Labdia Labordiagnostik, Vienna, Austria; 3 Department for Pediatric Oncology, Hematology and Clinical Immunology, Medical Faculty, Heinrich-Heine-University, Düsseldorf, Germany; 4 German Cancer Consortium (DKTK), partnering site Essen/Düsseldorf, Düsseldorf, Germany

**Keywords:** Cancer genetics, Acute lymphocytic leukaemia, Genetics research

## Abstract

Hyperdiploidy is the largest genetic entity B-cell precursor acute lymphoblastic leukemia in children. The diagnostic hallmark of its two variants that will be discussed in detail herein is a chromosome count between 52 and 67, respectively. The classical HD form consists of heterozygous di-, tri-, and tetrasomies, whereas the nonclassical one (usually viewed as “duplicated hyperhaploid”) contains only disomies and tetrasomies. Despite their apparently different clinical behavior, we show that these two sub-forms can in principle be produced by the same chromosomal maldistribution mechanism. Moreover, their respective array, gene expression, and mutation patterns also indicate that they are biologically more similar than hitherto appreciated. Even though in-depth analyses of the genomic intricacies of classical HD leukemias are indispensable for the elucidation of the disease process, the ensuing results play at present surprisingly little role in treatment stratification, a fact that can be attributed to the overall good prognoses and low relapse rates of the concerned patients and, consequently, their excellent treatment outcome. Irrespective of this underutilization, however, the detailed genetic characterization of HD leukemias may, especially in planned treatment reduction trials, eventually become important for further treatment stratification, patient management, and the clinical elucidation of outcome data. It should therefore become an integral part of all upcoming treatment studies.

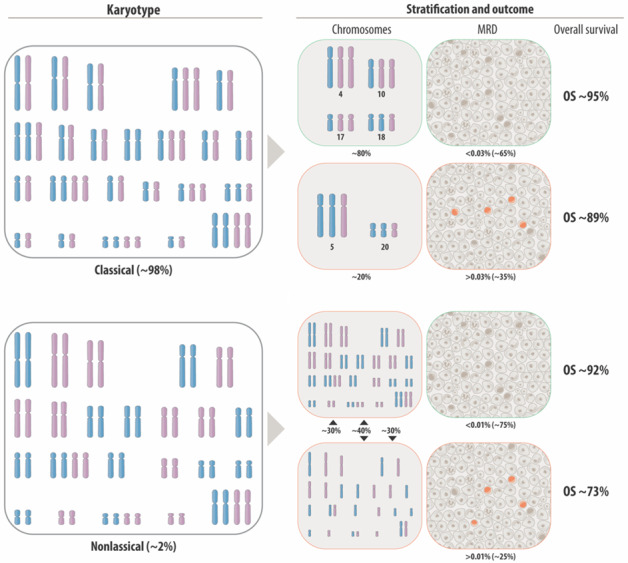

## Introduction

Hyperdiploidy (HD) was first described by Fritz Lampert in 1967 when he measured the DNA content of leukemic cells by meticulously comparing their diameters with those of normal lymphocytes [1]. Despite the rather crude cytogenetic methods that were available at that time, he also succeeded in quite accurately defining the nature of the acquired chromosomes and realized that the affected patients lived longer than others. Eventually, it became clear that a specific variant of HD is, with a prevalence of up to 35%, also the largest genetic entity of childhood B-cell precursor acute lymphoblastic leukemia (BCP ALL). Lampert’s remarkable insights have not only continued to prove accurate, but they have also become the subject of many research and clinical studies since then. Today, continuously evolving sophisticated diagnostic and research tools produce a plethora of information that unravels genomic peculiarities in previously unimaginable detail, and thereby helps to refine prognostic stratification and treatment.

Despite all these extraordinary achievements, many fundamental questions about the various aneuploid sub-forms of BCP ALL remain unresolved. These primarily concern their origins, the causes and biological meaning of the various nonrandom and disease-inherent chromosome configurations, and, not least, their varied disease development and clinical behavior. Although only a small proportion of the main HD variant experience relapses, it still makes up 25% of all relapses that occur in childhood BCP ALL. At present, the processes that drive these mainly late-disease recurrences are only vaguely understood [2].

Since the last comprehensive review of HD ALL by Paulsson and Johansson was published in 2009 [3], we consider it worthwhile to recapitulate the remarkable progress that has been made since then, especially in decoding the genomic and biological features of this extraordinary disease as well as in its prognostic stratification and treatment.

## Genomic features of HD ALL

### Chromosome copy number abnormalities

**Fig. 1:**
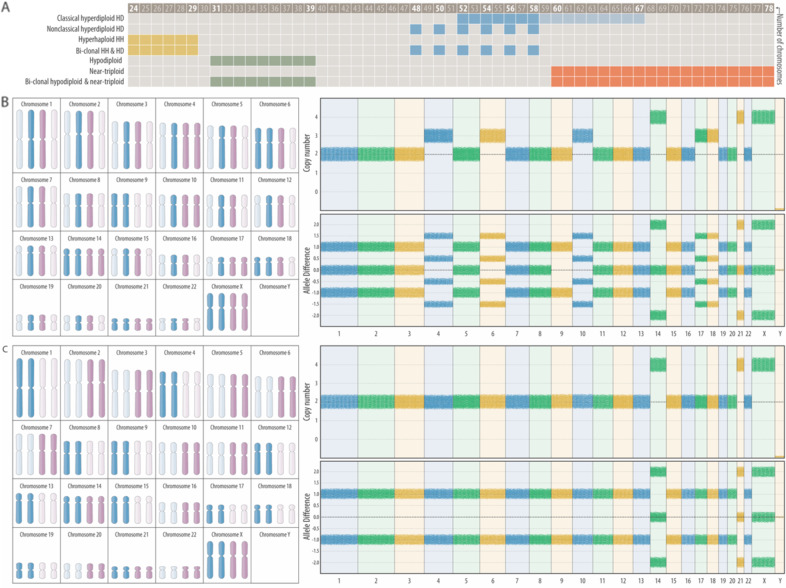
Defining criteria of classical and nonclassical HD forms of childhood ALL. **A** Based on their number of chromosomes, aneuploid forms of childhood ALL can be subdivided into seven distinct categories. The defining thresholds for HD cases range from 52 to 58 or 67 chromosomes. Classical HD and near-triploid karyotypes contain di-, tri, and tetrasomies, hyperhaploid and hypodiploid ones only mono- and disomies. Nonclassical HD karyotypes contain only di- and tetrasomies. 28% of the hyperhaploid and 32% of the nonclassical HD cases are monoclonal, whereas 40% of them share both clones. Likewise, 34% of the hypodiploid and 24% of the near-triploid cases are monoclonal, whereas 42% of them are bi-clonal [9]. The projection of representative karyotypes (highlighted chromosomes) of a classical (**B**) and a nonclassical (**C**) case onto the 92 chromosomes of a diploid mitotic cell illustrates that both patterns can be produced by the same yet-undefined nondisjunction mechanism and, in principle, even in a single step. The daughter cells that obtain the highlighted set of chromosomes, which always contain a tetrasomy 21, can survive, whereas the ones which only receive the dimmed set that lacks chromosomes 21 will perish as a result. The karyotype of the classical case (**B**) is 57,XX,+X,+X,+4,+6,CN-LOH(9),+10,+14,+14,+17,+18,+21,+21 and that of the nonclassical case (**C**) is 52,XX,+X,+X,CN-LOH(1–8,10–13,15–20,22),+14,+14,+21,+21.

Childhood ALL cases with gross ploidy changes are formally based on their DNA content and the overall number of chromosomes in their karyotypes. They comprise seven biologically related yet distinct categories whose common feature is a ploidy-related overrepresentation of chromosome 21, respectively (Fig. 1A). Although their categorization is primarily based on karyotype patterns, it takes increasingly also selected genomic features into account. Some of these new parameters are largely corroborating, whereas others uncover glitches and discrepancies in the current classification system, insights that justify a careful modification of the taxonomy as well as the terminology of these diseases that should take these novel findings into account [3–10]. Because the ploidy-related overrepresentation of chromosomes 21 serves as their essential and overarching hallmark, one can use the definitions of the “International System of Cytogenetic Nomenclature”, namely hyperhaploid (25–34 chromosomes), hypodiploid (35–45 chromosomes), hyperdiploid (47–58 chromosomes), hypotriploid (59–68 chromosomes) and hypertriploid (70–80 chromosomes) for their further subclassification without obscuring any otherwise pertinent information [11]. Just for convenience’s sake and practical reasons, the hypo- and hypertriploid may still be merged into a “near-triploid” group (59–80 chromosomes). In our review we will avoid the term “duplicated or doubled up haploids” because it insinuates a presumed but never proven mode that is supposed to generate this specific karyotype pattern. Since the more appropriate yet still not entirely correct descriptive term “hyperdiploidy due to a genome-wide loss of heterozygosity” would be rather impracticable to use, we opted to introduce the neutral terms “classical” and “nonclassical” for the two dissimilar but nevertheless closely related forms of genuine hyperdiploid forms that are the specific focus of our review. Both these types are defined by an agreed-upon chromosome number that lies on the lower side between 50 and 52 and on the upper side between 58 and 67, cutoffs that vary slightly in different studies (Fig. 1B, C). However, given that more than 80% of HD cases fall into the range of 52–58 with a modal peak of 55–56, we propose that an upper limit of 58 would be more appropriate, especially because such a cutoff eases the delineation of genuine HD forms from near-triploid ones (Fig. 1A) [4–8, 12–16].

The typical karyotype of the most prevalent classical HD variant is always composed of di-, tri-, and tetrasomies. Trisomies always result from the duplication of either one of the parental chromosomes in an apparently random fashion (“2+1” pattern) and most commonly affect chromosomes X, 4, 6, 10, 14, 17, and 18 (Fig. 1B) [3, 6]. Tetrasomies, on the other hand, always result from the duplication of both parental homologs (“2+2” pattern). The most common, in addition to the obligatory tetrasomy 21 are those of chromosomes X, 14, and 18. Although the individual chromosomal composition of the remarkably stable karyotypes varies from case to case, particular chromosomes appear in a predetermined, statistically hierarchical order [4, 6]. The probability that one or the other is seen depends on the chromosomes that are already present and, therefore, also on the overall modal chromosome number. Tetrasomy 21 is always the first change, which can then be followed, in a decreasing likelihood, by gains of chromosomes X, 14, 6, 18, 4, 17, and 10 [3, 6]. The composition of the various HD genomes is thus governed by the functional interdependence and indispensable compatibility of the respective combinations of chromosomes.

**Fig. 2:**
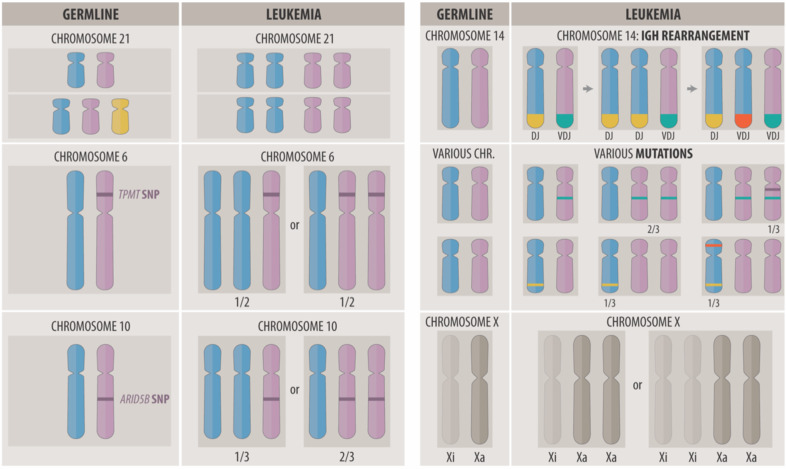
What the comparative analysis of germline and acquired chromosome copy number and/or sequence variants reveals about the origin, development, and biology of HD leukemias. Left side, top: the minimal common denominator of HD leukemia is always a bi-parental derived tetrasomy 21, irrespective of whether it arises in a constitutional normal or trisomic individual. Left side, middle: the duplication of either the wild-type or variant allele of pharmacologically relevant heterozygous genes, such as the thiopurine S-methyltransferase (TPMT) on chromosome 6 and/or the γ-glutamyl hydrolase (GGH) on chromosome 8, will produce two distinct leukemia genotypes with opposite drug sensitivities [34]. Left side, bottom: the *ARID5B* rs7090445-C risk allele is preferentially duplicated in HD blast cells [40]. Right side, top: the recombination of the immunoglobulin heavy chain (IGH) gene on chromosome 14 follows discrete consecutive steps during B-cell maturation. A clone with a disomy 14 can thus harbor a maximum of two unique rearrangements, whereas a clone with trisomy 14 can have three unique or one unique and two related rearrangements. Systematic analyses of such rearrangement patterns have shown that trisomy 14 is usually already present before the initiation of IGH recombination and thus prove that the maldistribution of the chromosomes is indeed the essential transforming event [162, 163]. Right side, middle: the analysis of acquired trisomy-associated heterozygous mutations informs about the sequence of events and the latency period between the nondisjunction event and their emergence. Mutations acquired before trisomy formation may affect either 2/3 or 1/3 of the duplicated homologs, whereas those that are acquired after trisomy formation can merely be present in 1/3 of the non-duplicated homologs [16, 55]. Right side, bottom: X inactivation (Xi) is a dosage-compensation mechanism in females that silences either the maternal or paternal chromosome with an equal likelihood during early fetal development. As in other HD-related trisomies, either one of the two parental X chromosomes can therefore be duplicated [164]. Notwithstanding this fact, however, it is always the active X (Xa) that is nonrandomly gained [165]. This outcome concords with the high expression of X-encoded genes and suggests that specific X-linked factors help to jumpstart and maintain the disease process [61–63].

The karyotypes of the second, much rarer nonclassical HD variant, contain only disomies and tetrasomies. This unique pattern was already recognized and described in the early days of cytogenetics (Fig. 1C) [17]. Array analyses revealed that the disomic chromosomes are always homozygous (“2+0” pattern), whereas the tetrasomic ones remain heterozygous (“2+2 pattern”) (Fig. 2). The same pattern is also seen in exceptionally rare cases with 48, 50, or 52 chromosomes. Notwithstanding their low chromosome numbers, however, we propose that they likewise belong to the nonclassical HD group. Although uniparental isodisomies may also appear in classical HD forms, they only involve a single or few chromosomes in those cases [18]. Nonclassical HD karyotypes are exact duplicates of hyperhaploid ones, and both can appear either alone or in combination. This circumstance led to the understandable but hitherto unproven view that nonclassical HD cases are merely duplicated hyperhaploid ones and that they can therefore be equated with hyperhaploidy, irrespective of whether a hyperhaploid clone is indeed identified or not [3, 7, 9, 19–25]. Thus, this uncritical synonymous use of these terms in this context is confusing, may often lead to misunderstandings, and, as we argue herein, it may probably also not be the correct label for how this hyperdiploid variant is formed.

The chromosome number of near-triploid cases lies between 60 and 78, which means that at least those with a chromosome number below 67, overlap with those classical HD ones with a chromosome number above 60 (Fig. 1) [7, 8, 13–15]. Because of the dissimilar clinical impact of classical HD and near-triploid cases, the proper assignment of such ambiguous cases to one or the other group is important for appropriate treatment stratification, a requirement that is, however, virtually impossible to fulfill based on chromosome counts alone [13]. Helpful karyotypic parameters that may delineate most of the near-triploid cases are the presence of a tri- or tetrasomy 1 together with a relative underrepresentation of chromosomes 7 and 14 [13]. Nevertheless, by far the best discriminators are either germline or somatic TP53 mutations, which are seen in virtually all near-triploid and hypodiploid cases, but hardly ever in classical or nonclassical HD ones (Fig. 1) [7, 8, 13–15].

Although constitutional trisomy 21 is by far the most common leukemia-predisposing factor, it is odd that classical HD forms are significantly underrepresented in this condition. To the best of our knowledge, none of the other aneuploid leukemia types have ever been reported in Down syndrome patients either [10, 26–28]. Yet, once formed, the karyotype patterns of such classical HD cases resemble exactly the ones, which are seen in constitutionally normal patients. In contrast to what one might intuitively expect, they never contain six chromosomes 21, but only the usual four or, as may occasionally be the case in constitutionally normal HD patients, five copies of chromosome 21. This suggests that a preexistent trisomy 21 impedes a priori the formation of the essential tetrasomy and/or that daughter cells with six chromosomes 21 cannot survive (Fig. 2) [10].

Of further interest in this context is DYRK1A, a serine/threonine kinase that is encoded on chromosome 21 and whose copy number determined overexpression is implicated in several pathologies in Down syndrome. Notably, this overexpression increases the expression and phosphorylation of two transcription factors, FOXO1 and STAT3, that are indispensable for B-cell development and therefore also contribute to the development and maintenance of BCP leukemias [29]. These effects are obviously especially pronounced in HD cases (https://pecan.stjude.cloud/proteinpaint/DYRK1A) and render both DYRK1A and FOXO1 worthwhile therapeutic targets in this specific subset of ALL [29].

Nonrandom secondary events in the form of structural abnormalities include chromosome 1q duplications (10–15%), 6q deletions (5%) and isochromosomes 17q (2–5%) and 7q (1–2%) [3]. They occur in a mutually exclusive manner and, may also be present in monoclonal nonclassical HD cases. However, to the best of our knowledge, they have never been observed in bi- or monoclonal hyperhaploid cases [5, 9, 10].

Another notable phenomenon is the co-occurrence of classical HD and an additional class-specific abnormality within the same cell clone, such as a t(9;22)(q34;q11)/*BCR::ABL1*, a t(1;19)/*TCF3::PBX1* or a t(4;11)/*KMT2A::AFF1* [22, 30, 31]. Either one of these can act as a primary or secondary change. In children, such “double hit” events comprise 2–3% of all HD cases, but they are 10–15 times more common in adults and therefore make up half of the total 13% of classical HD cases [31]. Although there are even a few mono- or bi-clonal nonclassical and/or hyperhaploid cases that concur with a t(9;22), we are not aware of any hypodiploid and/or near-triploid ones with such a constellation [28, 32, 33].

### Germline predisposing sequence alterations

Germline predisposition factors comprise genome-wide association study–ascertained allelic variants, which function as genetic modifiers, and distinct pathogenic gene defects (Table 1). An HD-inherent unique phenomenon is those heterozygous variants in pharmacologically relevant genes that are a priori disease-unrelated but nevertheless functionally important because their alternate allelic duplication will distort the concordance of germline and leukemia genotypes (Fig. 2) [34].

**Table 1:** Overview of susceptibility factors as well as predisposing germline and somatically acquired pathogenic genetic lesions that relate to classical and nonclassical forms of HD in childhood ALL (adapted from [10]).

Chromosome location	Candidate genes	Function	SNP/defect	Classical HD		Nonclassical HD and hyperhaploid	References
				Germline	Somatic	Somatic	
1p13.2	*NRAS*	RTK/RAS	PV		+	+	[7, 48, 53, 55, 56, 128, 129]
2p22.1	*SOS1*	RTK/RAS	PV	+			[48]
3p21.31	*SETD2*	Histone methyltransferase	PV		+		[7, 55, 56]
4p16.3	*NSD2 (WHSC1)*	Histone methyltransferase	PV		+		[53, 55]
5p14.2	*PRDM9*	Histone methyltransferase, meiotic recombination	Rare allele	+			[130, 131]
5q31.1	*C5orf56*	lncRNA	rs886285	+			[132]
6p21.31	*BAK1*	Pro-apoptotic BCL2 antagonist	rs210143	+			[132]
6p22	Histone cluster	DNA replication and packaging	Deletions		+	+	[7]
6q22.1	*ROS1*	RTK/RAS	PV		+		[56]
7p12.2.	*IKZF1*	Lymphoid transcription factor	Deletions		+	+	[2, 7, 52, 129, 133]
7q36.1	*EZH2*	Histone methyltransferase	PV		+		[7, 55, 56]
8q21.13	*PAG1*	Ras signaling inhibitor	Deletions		+	+	[7]
9p21.3	*(TLE1)*	Transcription co-repressor	rs76925697	+			[132]
9p21.3	*CDKN2A* and *CDKN2B*^a^	Cyclin-dependent kinase inhibitors	rs3731217, rs77728904	+			[134–136]
9p21.3	*CDKN2A* and *CDKN2B*^a^	Cyclin-dependent kinase inhibitors	Deletions^b^		+	+	[52, 132, 134, 135]
10p12.2	*BMI1*	Chromatin remodeling epigenetic repressor	rs7088318	+			[36, 37]
10q21.2	*ARID5B*	Histone demethylase, transcription coactivator	rs7090445	+			[35, 40, 137, 138]
10q21.2	*ARID5B*	Histone demethylase, transcription coactivator	Deletions		+		[139]
10p12.2	*PIP4K2A*	PI-3-kinase regulator	rs2296624	+			[36, 37]
11q13.2	*KMT5B (SUV420H1)*	Transcription regulator, histone methyltransferase	PV		+		[7, 55]
11q14.1	*GAB2*	PI-3-kinase regulator	PV	+			[140]
12p12	*KRAS*	RTK/RAS	PV		+	+	[7, 48, 53, 55, 56, 128]
12p13.2	*ETV6*	ETS transcription factor, transcription repressor	Multiple SNPs	+		+	[47, 49]
12p13.2	*ETV6*	ETS transcription factor, transcription repressor	PV, deletions^b^	+^c^	+	+	[47, 49, 50, 141–143]
12q24.1	*PTPN11*	RTK/RAS	PV	+^d^	+	+	[48, 53, 55, 56]
13q12.2	*FLT3*	RTK/RAS	PV, duplications		+	+	[7, 48, 53, 55, 56, 144, 145]
13q12.2	*PAN3*	RTK/RAS	Deletions		+^e^	+	[146]
13q14.2	*RB1*	Transcription factor, negative cell-cycle regulator	PV, deletions		+		[7, 129]
14q11.2	*CEBPE*	Transcription activator	rs2239630	+		+	[35, 40, 132, 138, 147]
16p13.3	*CREBBP*	Transcription coactivator	PV, deletions	+^f^	+	+	[52, 53, 55, 56, 129]
16q22.1	*CTCF*	Zinc finger transcriptional repressor	PV, deletions		+		[57]
17q11.2	*NF1*	RTK/RAS	PV, deletions	+	+	+	[7, 55]
17q21.1	*IKZF3*	Lymphoid transcription factor	Haplotype	+		+	[147]
17q21.1	*IKZF3*	Lymphoid transcription factor	Deletions		+		[7]
19p13.3	*DOT1L*	Histone methyltransferase	PV		+		[56]
21q22.2	*ERG*	ETS transcription factor, transcription regulator	rs9976326	+			[132]

Most of the listed somatic mutations were also identified in the recent study by Brady et al. in 324 hyperdiploid and 24 hyperhaploid ALL cases [16].

*PV* pathogenic variants, *SNP* single-nucleotide polymorphism, *RTK/RAS* component of the receptor tyrosine kinase signaling pathway.

^a^Generic B and T ALL predisposing SNPs.

^b^Common in virtually all types of B and T ALL.

^c^*ETV6*-linked leukemia predisposition and familial thrombocytopenia syndrome.

^d^Noonan syndrome-type Rasopathy.

^e^*FLT3* upregulation via *PAN3* deletion-associated enhancer hijacking.

^f^Rubinstein–Taybi syndrome.

The four most relevant susceptibility loci reside within or in close proximity to the *ARID5B*, *CEPBE*, *BMI1*, and *PIP4K2A* genes [35–41]. ARID5B plays an essential role in the epigenetic activation of gene expression, cell-cycle regulation, and 6-mercatopurine and methotrexate (MTX) metabolism [37, 40, 42, 43]. The intronic rs7090445-C risk allele of this gene is less expressed than that of the wild-type one. Functional studies and analyses of many carriers have shown that its presence will impede normal lymphocyte development, facilitate the clonal expansion of the affected BCPs, confer drug resistance, and increase the relapse risk [37, 40, 42–46]. *CEBPE* encodes one of six basic leucine transcription factors. The risk-defining SNP rs2239635 in its promotor disrupts the binding of the Ikaros transcriptional repressor [39]. *CEBPE* is on chromosome 14, which is incidentally also one of the most common tri- and tetrasomic chromosomes in HD. Its copy-number-linked overexpression may thus be one of the critical contributing leukemia-promoting factors [39]. BMI1 is a negative regulator of the cell-cycle checkpoint proteins p16 and p14ARF, encoded by *CDKN2A*, which is the most frequently deleted gene in ALL. The risk-defining SNP rs11591377 is in a predicted hematopoietic stem cell enhancer and reinforces the preferential binding of the MYBL2 and p300 transcription factors [41]. *PIP4K2A* encodes an enzyme that is part of the phosphoinositide signal transduction pathways that co-regulate cell proliferation, differentiation, and motility. The risk-defining SNP rs4748812 lies within a *PIP4K2A* regulatory element and is predicted to alter the binding of the RUNX1 transcription factor [41].

A not yet exactly determined proportion of HD ALL cases have predisposing pathogenic germline defects in genes that encode members of B-cell development, receptor tyrosine kinase/RAS (RTK/RAS), epigenetic regulatory, and DNA repair pathways (Table 1). The two most common conditions concern the *ETV6* and the *PTPN11* genes [16, 47–49]. Approximately 70% of all BCP ALL cases with germline defects in either one of these two genes are hyperdiploid [48, 49]. *ETV6* germline-mutated cases frequently also acquire somatic mutations in *NRAS*, *KRAS*, and *PTPN11* [49]. Although we have no specific information about secondary changes in cases with RASopathy, it is worth noting that in two of them the *PTPN11* mutation became duplicated in the form of a uniparental disomy 12 [48].

Predisposing germline factors are unlikely to be directly responsible for the formation of HD precursor cells per se but rather alleviate the immediate survival of independently created cells by equipping them with essential elements that founder cells in non-predisposed individuals are forced to acquire as secondary changes [10]. The extent to which the occasional concurrence of two or even more such predisposition factors, for instance, *ARID5B* and *ETV6*, will augment the respective risk remains to be determined [50].

### Somatically acquired sequence alterations

Virtually all classical and nonclassical HD cases also acquire somatic mutations, primarily in the RTK/RAS and phosphoinositide 3-kinase-signaling pathway genes *KRAS*, *NRAS*, *FLT3, SOS*, and *PTPN11*, as well as in the chromatin-modifying genes *CREBBP*, *NSD2*, *SUV420H1*, *SETD2*, and *EZH2* (Table 1) [16, 51–57]. Other abnormalities that are typically enriched in nonclassical HD forms are *NF1*, *CDKN2A/B*, *IKZF3*, *PAG1*, and the 6p22 histone gene cluster [7, 22]. Most of these genes are located on chromosomes that usually remain disomic. Although the frequency of mutations is much higher in disease recurrences, the originally extraordinary inter- and intragenic heterogeneity of mutually exclusive RTK/RAS pathway mutations is then essentially lost. *KRAS* mutations are the only ones that are preferentially retained and then commonly coexist with the prevalent *CREBBP* mutations in the predominant relapse clones [52, 53, 58].

Of note in this context are the comparative analyses of the numbers of monoallelic and biallelic mutations on disomic and on trisomic chromosomes, which revealed that monoallelic mutations on trisomic and homozygous ones on disomic chromosomes are significantly more common than biallelic ones. These observations provide compelling evidence that such sequence alterations emerge only quite some time after the formation of the hyperdiploid genome (Fig. 2) [16, 55].

### Mutation signatures

Mutation signatures can reveal environmental and endogenous sources of mutagenesis in affected tissues. At present, there are 49 accepted single base substitution signatures, whose causative factor has been established with a certain security [59, 60]. Of these, Signature7 is the one that is induced by exposure to ultraviolet light [59, 60]. Brady et al. found this signature especially enriched in 17% of hyperdiploid, 35% of hyperhaploid as well as 46% of iAMP21-positive leukemias exclusively in patients of non-African descent [16]. The fact that these mutations emerged only after the aneuploidization event had taken place was therefore taken as an indication that it can only be postnatally induced when the respective cells become trans-dermally exposed to skin-penetrating light. As interesting as these intriguing observations together with the interpretation of their emergence are, they certainly need to be further scrutinized and functionally evaluated [59].

### Methylome, transcriptome, and proteome

The methylation, gene expression, and protein structure of HD leukemias are tightly interlocked and mainly shaped by dosage effects that are exerted by the surplus chromosomes [30, 61–68]. Compared to other types of ALL, HD leukemias are remarkably hypomethylated, a feature that remains constant regardless of the number and types of chromosomes that are present in the individual karyotypes. This observation has therefore been taken as an indication that this peculiar signature must either predate or at least concur with the acquisition of the extra set of chromosomes [68]. The six different pathways that are enriched in the expression signature of HD ALL include translation and ribosomes, innate immunity, cell adhesion, cytokines and activated signaling, protein folding and proteolysis, and the endosome pathway [63, 69]. Although the expressed genes and the associated range of proteins largely correlate with the number of chromosomes, the overall effect is nevertheless determined by those almost 70% of genes on chromosomes 21, X, and 14 [30, 61–63, 65]. Conversely, approximately 16% of the transcript and 25% of the protein levels are significantly lower than their corresponding gene copy number would indicate [63]. The top-downregulated genes and proteins are IGF2BP1, CLIC5, RAG1, and RAG2 [63]. Such a diminished *IGF2BP1* and *CLIC5* gene expression is one of the outstanding features of HD leukemias with CTCF alterations and histone gene cluster 1 deletions [57]. The transcriptional repressor CTCF and the cohesion complex are not only master regulators of the chromatin architecture but also of transcription [63, 70]. By binding to chromatin insulators, CTFC prevents the interaction between promoters and nearby enhancers and silencers. Its encoding gene, *CTFC*, is on chromosome 16, which usually remains disomic, whereas the core members of the cohesion complex are on trisomic chromosomes (*RAD21* on 8q24, *SMC3* on 10q25, *SMC1A* on Xp11, and *STAG2* on Xq25). It was therefore proposed that this copy number discrepancy may likewise unbalance the essential expression equilibrium between these genes [63]. Although CTCF depletion and cohesin loss will first of all impair the proper cohesion, alignment, and segregation of chromosomes, it will also weaken the insulation at the borders of topologically associating domains [63, 69–71]. Since these chromatin structures control the timing of DNA replication, the destruction of their framework has also a severe effect on the genome-wide coordination of gene expression [63, 72]. Together with the faulty activity of Aurora B kinase and Survivin, which normally fine-tune the spindle assembly checkpoint, this genomic turmoil slows down the mitotic process, decreases the proliferative rate of HD cells, and is also responsible for the poor morphological appearance of metaphase chromosomes, which often makes them extremely difficult to analyze. Since neither cohesin nor condensin complex encoding genes are consistently mutated in HD leukemias, these effects can only be caused by the subordinate inadequate expression and resultant dysfunction of cohesin and/or condensin complex components [69, 71].

Notably, the gene expression profiles of classical and nonclassical HD forms are virtually identical. In unsupervised hierarchical clustering and principal component analyses, monoclonal nonclassical HD, bi-clonal, and monoclonal hyperhaploid entities form a single discrete cluster that clearly separates them from the hypodiploid/near-triploid entities, which, in turn, form their own cluster [7]. Moreover, classical and nonclassical HD forms even cluster together in an indistinguishable manner in a t-distributed stochastic neighbor embedding blot analysis [16, 73].

Finally, we need to mention the copy-number-related overexpression of the *SLC19A1* gene on chromosome 21q22.3, which encodes the predominant folate and MTX uptake transporter [74, 75]. The larger amount of this transporter also increases the quantity of polyglutamates, the active metabolites of MTX and mercaptopurine, as well as thioguanine nucleotides, in blast cells. The presence of a tetrasomy 21 is therefore thought to explain, in part, the good outcome of patients with HD leukemias [76–80].

## The roots of HD leukemias

HD leukemias evolve from a single immature BCP cell, which is transformed very early during fetal development [81–89]. Although there is general agreement that the initiating event is a flawed cell division during which the leukemic precursor cell is supposed to receive all extra chromosomes instantaneously, the actual cause of the triggering nondisjunction error is currently still unknown [3, 16, 90]. Since decades of extensive research have not uncovered any potentially responsible genetic defect, we proposed that the critical nondisjunction and segregation errors may be due to a physical disruption of the intricate spindle scaffold of a mitotic cell, which could be due to the untimely cytoplasmic influx from a second, partially fused interphase cell [10].

The current and intensely scrutinized view suggests that a nonclassical HD karyoytpe can only derive from the duplication of a previously generated hyperhaploid one [3, 7, 8, 14]. However, as we show in Fig. 1, it may indeed be more likely that the nonclassical HD clone is generated first in a similar fashion as the classical HD one. Reversing the order of appearance of nonclassical HD and hyperhaploid clones, simplifies the entire concept of how classical, nonclassical, and hyperhaploid HD cases are interrelated. If the nonclassical HD clone comes first, the hyperhaploid clone can only be its descendant, and then, may either coexist as a secondary change or even outperform the original nonclassical HD clone [10]. Another albeit more remote alternative possibility could be that the hyperhaploid clone originates from the cell that is supposed to cause the nondisjunction error [10].

## Clinical and biological features

In the western world, up to 35% of childhood leukemias are hyperdiploid, but with 15–25%, they are far less prevalent in patients of Asian, African, and Native American descent [91–93]. Children with HD ALL are young (median age of about 4 years). At diagnosis, they have a white blood cell count that is typically below 10^9^/l and no extramedullary disease. HD blast cells show a high expression of CD9, CD20, CD22 CD58 CD 66c, CD86, and CD123, and a low expression of CD45 [94]. Their most relevant immunophenotypic feature is the aberrant expression of CD123, the interleukin-3 receptor alpha chain, which is encoded in the pseudo-autosomal regions on Xp22.3 and Yp11.3 [95–97]. This marker is therefore also commonly used as an indicative surrogate flow-cytometric predictor of classical HD leukemias.

One thought-provoking discovery is the fact that heterozygous HLA-DPB1*0201 alleles are significantly enriched in patients with HD leukemias [98]. HLA-DPB1*0201 belongs to the HLA class II genes that are important in adaptive immune responses to infection. This preponderance has therefore been taken as evidence that yet-undefined intrauterine immunological processes execute selective forces and mediate a proliferative stress on preleukemic HD cells, effects that may then again be somewhat mitigated by protective life-style factors, such as breastfeeding and day-care attendance [45, 99, 100]. Supporting these HLA data are now recent epidemiological data and laboratory findings. They revealed that prenatal cytomegalovirus infections of the patients’ mothers are probably one of the most relevant etiologically factors for initiating and/or promoting especially the development of HD leukemias [101–103]. Moreover, this hazard is particularly pronounced in carriers of an *ARID5B* risk allele [101–103].

HD ALL blast cells are inherently difficult to maintain and propagate in culture, which makes it extremely difficult to perform not only cytogenetic analyses, but also any other type of research that depends on viable cells [104]. Underlining this problem is the fact that there are no established cell lines from patients with HD leukemias, and only one that derives from a nonclassical HD (MHH-CALL-2) and a second from a hyperhaploid case (NALM-16) [3, 25, 105–107]. The only way to maintain and propagate HD blasts is therefore to either cultivate them on autologous feeder layers or to xenograft them [104, 108].

## Diagnostic assessment, disease stratification, and treatment outcome

Table 2 summarizes the pros and cons of the various technologies that are instrumental not only for the identification and delineation of HD ALL cases, but also for the in-depth analyses of their genomic structure. Several of these technologies are quite sophisticated, so it is quite surprising that those being used almost exclusively for treatment stratification today are still the rather basic options that have been already in use for many decades. The reason for this is that these diagnostic tools are simple to perform, cheap, and fast [109–112]. However, the fact that they are still considered sufficient also implies that, for clinical purposes, a more in-depth evaluation of the intricate features of such cases is deemed completely unnecessary, since these patients have in any case an overall very good outlook (Table 3). Yet, as alluded to above, this tactic does certainly neither suffice for the demarcation and clear assignment of classical and nonclassical HD cases nor for that of classical HD and near-triploid ones, respectively. Such delineation ambiguities can to a certain extent obscure the outcome results that are obtained by different treatment studies. Moreover, applying the usual DNA content of equal to or more than 1.16 (equivalent to approximately 54 chromosomes) as the lower defining HD threshold will fail to secure cases with karyotypes that contain only a smaller and/or lower number of chromosomes. As pointed out by Carroll et al., such allocation problems cause the misclassification of a considerable proportion (at least 25%) of nonclassical HD cases [9]. They showed that studies that evaluated the treatment outcome of mono- and bi-clonal hyperhaploid cases never included monoclonal nonclassical HD cases, even though they are always equated with hyperhaploid ones and automatically assigned to the high-risk group, in case they are indeed identified [5, 9, 113–115]. Carroll’s observation can only mean that monoclonal nonclassical HD cases were either recognized but purposely not included or not recognized and consequently stratified and handled the same way as classical HD cases.

**Table 2:** Advantages and disadvantages of various diagnostic technologies for the diagnostic assessment of HD ALL.

Method	Advantages	Disadvantages
DNA index measurement	• Fast, cheap, and easy • Can to a certain extent identify multiple abnormal clones to a certain extent	• Provides only a rough estimate of the DNA content but no information about chromosomal composition of the respective clones • Cannot differentiate between classical and nonclassical HD forms
Cytogenetics	• Provides information about types and numbers of chromosomes	• Relies on the availability of blast cell metaphases • Information derives only from single dividing cells
Fluorescence in situ hybridization (FISH)	• Allows interphase screening • Provides information about composition and size of cell clones	• Utilized to screen for selected relevant chromosomes only • Cannot elucidate the chromosomal composition of the entire genome
CGH/SNP array analysis	• Provides a representative genome-wide profile of all large- and small-scale copy number alterations as well as their allelic pattern	• Cannot distinguish between monoclonal and/or bi-clonal forms of hyperhaploid and nonclassical HD forms
Whole-exome sequencing (WES)	• Provides exome-wide information about copy number as well as sequence alterations	• Primarily a research tool • Mutations hardly diagnostically or therapeutically relevant in HD
Whole-genome sequencing (WGS)	• Provides genome-wide information about all relevant copy number, structural, and sequence alterations simultaneously • Could replace all other methods, in principle	• Cannot distinguish between monoclonal and/or bi-clonal forms of hyperhaploid and nonclassical HD forms • Not yet implemented for routine diagnostics
Gene expression (GEP) analysis	• Reflects chromosome copy number changes • May be utilized for mutation calling • Can identify fusion genes • Uncovers the close relationship between mono- and/or bi-clonal nonclassical HD and hyperhaploid forms	• Diagnostically not relevant • Primarily a research tool
Optical genome mapping	• Provides genome-wide information about copy number and structural abnormalities • Reveals allele-specific patterns • May eventually replace karyotype, FISH, and array analyses	• Research tool only • Not yet implemented in the diagnostic work-up • Cannot detect sequence variants

**Table 3:** Standard risk stratification and outcome of children with HD ALL in contemporary treatment protocols.

Study protocol	Age (years)	WBC ×10^9^/L	CNS status	Treatment response					Genetic parameters	Outcome	Ref.
				Steroid response^a^	Day 8	Day 15	End of induction	Later timepoints			
AIEOP-BFM 2000 ALL	1–17	Any	Any	+	<1.000 blast/µl (PB)	n.a.	PCR MRD negative	Day 78 PCR negative	–	Standard DI PII: 4-year OS 97.9% 8-year DFS 92% reduced intensity DI PIII: 4-year OS 93.4% 8-year DFS 90%	[148, 149]
ALL IC-BFM	1–6	<20	Any	+	<1.000 blast/µl (PB)	M1/M2	–	–	–	5-year EFS 81% 5-year OS 90% (all SR patients combined)	[150]
CoALL 07–03	<10	<25	Any	–	–	MRD <0.01%	MRD <0.01%^b^	–	–	Number of identified HD patients is too low	[151]
COG AALL 0331 LRS	1–10	<50	CNS1 only	–	M1^c^	M1^c^	MRD <0.01%^b^	–	+4, +10, +17	6-year CCR 96.3% 6-year OS 99.4%	[116, 118]
COG AALL 0932 LR-C	1–10	<50	CNS1 only	–	MRD <0.01% (PB)	–	MRD <0.01%^b^	–	+4, +10	5-year EFS 98.8% 5-year OS 100% (all LR patients combined)	[117]
COG AALL 0932 LR-M	1–10	<50	CNS1 only	–	MRD <0.01% (PB)	–	MRD <0.01%^b^	–	+4, +10	5-year EFS 98.5% 5-year OS 100% (all LR patients combined)	[117]
COG P9904	1–10	<50	No CNS3	–	MRD <0.01% (PB)		MRD <0.01%^b^	–	+4, +10	5-year EFS 97% 5-year OS 98% (all LR patients combined)	[117]
RELLA05	1–9	<50	CNS1/CNS2 or traumatic LP		–	MRD <0.01%^d^	–	–	DNA index >1, 16	5-year EFS 92% 5-year OS 96% (LR group combined)	[152]
JACLS-ALL-02	1–9	<10	No CNS3	+	<1.000 blast/µl (PB)	M1/M2 BM	M1 BM	–	–	4-year EFS 89.4% 4-year OS 94.9%	[153]
JACLS-ALL-02 SR	1–9	<10	No CNS3	+	<1.000 blast/µl (PB)	M1/M2 BM	M1 BM	–	–	4-year EFS 100% (+4, +10, +17) 4-year EFS 88.2% (other HD)	[93]
St. Jude Total XVI	1–10	<50	Any	–	–	MRD <1%	D46 MRD <0.01%	–	DNA index >1, 16	5-year EFS 95.3% 5-year OS 99.4%	[154, 155]
UKALL 2003	1–10	<50	Any	–	–	M1/M2 BM	MRD <0.01%	–	–	10-year survival: all HD patients 90% EFS, 94% OS good risk HD 92% EFS 96% OS poor risk HD 81% EFS, 86% OS	[120, 156]
UKALL 97/99	1–10	<50	Any	–	–	M1/M2 BM	–	–	–	10-year survival: all HD patients 84% EFS, 93% OS good risk HD 86% EFS, 94% OS poor risk HD 71% EFS, 89% OS	[120]
NOPHO ALL 1992/2000	2–10 (1992) 1–10 (2000)	<10	CNS1	–	–	M1/M2 BM	M1 BM	–	Karyotype (central review)	5-year EFS 82% 10-year EFS 80% 5-year OS 91% 10-year OS 89% (both studies combined)	[157, 158]
DCOG 10	1–18	Any	CNS1 only	+	<1.000 blast/µl (PB)	–	PCR negative	Day 80 PCR negative	–	5-year EFS 87.9%, 5-year OS 93.3%	[159]
CCLG-ALL 2008	1–10	<50	No CNS3	+	<1.000 blast/µl (PB)	M1/M2 BM	PCR negative or flow MRD <0.01%	Week 12 PCR negative or flow MRD <0.01%	–	5-year EFS 81.6% 5-year OS 91.5% (SR group combined)	[160]
TCCSG L04–16	1–6	<20	No CNS3	+	<1.000 blast/µl (PB)	–	Day 43 M1	–	–	5-year EFS 85% 5-year OS 95.4%	[161]

*CNS* central nervous system involvement, *PB* peripheral blood, *BM* bone marrow, *MRD* measurable residual disease, *DFS* disease-free survival, *ES* event-free survival, *OS* overall survival, *CCR* continuous complete remission, *PII/PIII* protocol II or protocol III of AIEOP/BFM, respectively, *DI* delayed intensification.

^a^Patients were exposed for 1 week to prednisone monotherapy before chemotherapy on day 8 has been initiated. On day 0, one additional dose of methotrexate was given intrathecally. The presence of less or more than 1.000 blasts/μl PB on day 8 was defined as prednisone good or poor response, respectively.

^b^Day 29 responses.

^c^Day 8 or day 15 M1 bone marrow.

^d^Day 19 MRD <0.01%.

+ day 29 MRD detectable but <0.01% and undetectable before start of interim maintenance.

The two central challenges that clinicians are nowadays confronted with in HD leukemias are how to identify patients with a high propensity to relapse already at diagnosis and how to reduce treatment in low-risk patients without jeopardizing their good outcome. Virtually all contemporary, ongoing, and planned treatment studies rely on the assessment of the measurable residual disease (MRD), either based on the PCR-based quantification of immunoglobulin and T-cell receptor gene rearrangements or based on the flow-cytometric assessment of the immunophenotypic criteria of blast cells. Only some of these studies also require the identification of HD cases, which is then achieved by determining the DNA content, the overall chromosome number and/or the copy numbers of selected chromosomes with fluorescence in situ hybridization (FISH) that are considered appropriate for the delineation of good risk cases. Such a chromosomal risk classification has, for instance, already been used successfully in the Children’s Oncology Group study for over 25 years (Table 3) [116–118]. O’Connor et al. and Enshaei et al. have recently succeeded in significantly refining and improving the prognostic value of this genetic stratification system. First, they reported that at least in the UKALL treatment studies, the optimal MRD threshold for HD cases, which derives from a retrospective statistical analysis of data, is 0.03% rather than the 0.01% cutoff that is normally used [119, 120]. This elaborated MRD threshold of 0.03% for HD cases will now be prospectively evaluated in the newly established ALLTogether consortium, which comprises the Nordic countries, Estonia, Lithuania, the United Kingdom, the Netherlands, Belgium, Ireland, and France (https://clinicaltrials.gov/ct2/show/NCT03911128). Whether such a challenging detailed threshold definition is indeed also technically feasible to routinely achieve and therefore worthwhile to implement in future clinical studies, remains to be seen. Second, Enshaei et al. convincingly proved that the copy number assessment of four chromosomes, whose prognostic relevance was already appreciated previously is sufficient to demarcate two distinct risk groups. The low-risk group, which in their study makes up 80%, is defined by trisomies 17 and/or 18, whereas their poor risk group makes up 20% and is defined by trisomies 5 and 20 [120]. This poor risk group includes nearly half of the relapse cases. Since trisomies 5 and 20 are rarely seen in cases with less than 58 chromosomes [121], it will be important to evaluate to which extent their prognostic value depends on or is influenced by the overall chromosome number. Nevertheless, the respective UKALL profile still outperforms other trisomy-based ones and is independent of the results of MRD measurements, although it might be expected that combining both will increase the value of this risk score even further. Especially in the context of the planned treatment reduction for low-risk HD cases, we consider it appropriate to further substantiate these findings in prospective studies of large cohorts of genetically thoroughly defined HD cases.

Of further note in this context is the recent observation that nonclassical HD cases with *SETD2* mutations had an inferior event-free (8/280 cases; 47% versus 95%) and overall survival than nonaffected ones [16].

Owing to the lack of more refined genetic discriminators, all mono- and bi-clonal hyperhaploid and nonclassical HD forms are hitherto stratified as high risk. However, Mullighan et al. showed that even in those cases MRD is the most important prognostic indicator since all cases with a negative MRD status (<0.01%) are highly curable with intensive chemotherapy alone [54].

The high cure rates of low-risk classical HD ALL cases imply that many of them are probably overtreated. The daunting challenge is now to reduce treatment intensity to diminish side effects and avoid ensuing long-term sequelae without endangering the hitherto achieved excellent overall treatment outcome. Some of the trials that have addressed this issue so far have been quite successful (even without utilizing any genetic risk score), whereas those trials which reduced the duration of maintenance therapy to 6 months were not, at least as regards HD ALL [55, 117, 122–125]. Although Kato et al. found that one year of maintenance therapy is probably sufficient for *TCF3::PBX1-* and *ETV6::RUNX1-*positive cases, it is definitely not adequate for all HD leukemias. As a result of relapses, their disease-free survival was only 56.6 ± 10.3% [123]. Nevertheless, viewed in reverse a remarkable proportion of patients also benefited; most relapses were salvageable, and the overall survival of the entire cohort still reached 91.7 ± 5.6% after 12 years [123].

The emergence of specific mutations in disease recurrences suggests that many of them are treatment induced. These mutations comprise, for instance, those in *NR3C1/2*, *CREBBP*, and *WHSC1* for glucocorticoids; *NT5C2*, *MSH2/6*, *PMS2*, and *PRPS1/2* for thiopurines; and *FPGS* for MTX [126]. Examining such mutation patterns in 103 ALL germline/diagnosis/relapse trios, Li et al. identified two novel relapse-specific signatures in 25% of children with an early or late relapse [126]. They were able to prove that one of these signatures was caused by thiopurine treatment, whereas for the other, which was significantly enriched in HD leukemias, they could not ascertain the respective cause.

Together these observations confirm that an adequate length of maintenance therapy is essential for preventing early relapses in classical HD leukemias but also, that this treatment component cannot be held accountable for inducing late ones, as seems to be the case in other genetic subtypes.

## Summary

HD leukemias have puzzled and confused researchers and clinicians for more than 50 years now. Novel findings that derive from array, mutation, and gene expression analyses offer now new opportunities to re-examine deeply engrained, yet largely unproven and unquestioned views about their origin, their mode of creation, and the interrelatedness of the diverse aneuploid sub-forms. Such fresh insights will eventually help to reconsider and refine the current classification system and thereby also influence the prognostic stratification and treatment of these subgroups. One example concerns the elucidation of the biological relatedness of classical, nonclassical (“duplicated hyperhaploids”), and pure hyperhaploid cases, which will help to better understand what drives their apparent different clinical behaviors. Another one concerns hyperdiploid forms with a high chromosome number. Near-triploid cases as well as mono- and bi-clonal hypodiploid cases can nowadays be easily ascertained, because over 90% of them are *TP53* mutated. However, it is less clear whether the remaining *TP53* wild-type ones with a similar high chromosome count should be also stratified as near-triploid or rather as classical hyperdiploid ones. Although this concerns only a few cases, their diagnostic clarification, and most appropriate allocation requires further scrutiny. Array analyses deliver not only a detailed information about copy number changes, but also about allele distribution alterations, such as the presence of otherwise unidentifiable uniparental disomies. Bearing the ongoing trend to reduce treatment for very low-risk patients in mind, which especially also concerns HD leukemias, we advocate to implement this state-of-the-art technology together with mutation screening in treatment studies to comprehensively characterize the detailed genomic make-up of HD leukemias [127]. Only such an approach, rather than, as is current practice, merely typifying them with DNA content measurements, cytogenetics, and/or selected FISH probes, can better our understanding of the biology and the clinical behavior of the karyotypically heterogeneous subsets of classical and nonclassical HD leukemias as well as oversee in a more individual fashion the effects of various treatment interventions. The invaluable results that have already been obtained with such thorough analyses in selected yet still less well-characterized cohorts clearly prove, that the study-wide implementation of such a policy will significantly foster basic as well as clinical research in this particular group of patients and thereby provide benefits for their management that will go far beyond the simple identification and prognostic grading of HD cases, as is still done today.
